# Obacunone inhibits microglia‐mediated neuroinflammation and ischemic injury by targeting MAPK1

**DOI:** 10.1002/alz70859_101787

**Published:** 2025-12-25

**Authors:** Xiaolei Zhu, Jing Huang, Yun Xu

**Affiliations:** ^1^ Affiliated Drum Tower Hospital of Nanjing University Medical School, Nanjing China; ^2^ Affiliated Drum Tower Hospital of Nanjing University Medical School, Nanjing, Jiangsu China; ^3^ Nanjing Drum Tower Hospital Clinical College of Nanjing Medical University, Nanjing, Jiangsu China

## Abstract

**Background:**

Microglia‐mediated neuroinflammation plays a critical role in the pathogenesis of ischemic stroke and represents as a promising target for stroke treatment. Obacunone (OB), a triterpenoid limonin extracted from natural citrus plants, exhibits anti‐inflammatory, antioxidant and anti‐cancer properties.

**Method:**

Primary microglia were pre‐incubated with OB for 2 hours, followed by LPS stimulation for 24 h or OGD treatment for 4 hours. Real‐time quantitative PCR, ELISA and western blot were performed to examine the expression levels of inflammatory cytokines. The mice were injected intraperitoneally with OB or vehicle after MCAO. Neurobehavioral tests and TTC staining were conducted to estimate the neurological deficits and infarct area. The white matter integrity after MCAO was observed via immunofluorescence staining and memroy fuctions were examined by new object recognition and MWM tests.

**Result:**

We demonstrated that OB inhibit the activation of the transcription factor nuclear factor κB (NF‐κB) pathway and reduced microglia‐mediated inflammatory cytokine production both in vitro and in vivo. Furthermore, OB attenuated ischemic brain injury and cognitive impairment in mice subjected to middle cerebral artery occlusion (MCAO). Network pharmacology analysis identified mitogen‐activated protein kinase 1 gene (MAPK1/ERK2) as a potential target of OB, which was verified by the binding assays.

**Conclusion:**

Our study suggest that OB alleviated neuroinflammation and ischemic injury, positioning it as a promising therapeutic agent for the treatment of ischemic stroke.

